# ﻿Three new asexual *Kirschsteiniothelia* species from Guizhou Province, China

**DOI:** 10.3897/mycokeys.113.139427

**Published:** 2025-02-03

**Authors:** Xing-Juan Xiao, Ning-Guo Liu, Jian Ma, Li-Juan Zhang, Dan-Feng Bao, Song Bai, Fatimah Al-Otibi, Kevin D. Hyde, Yong-Zhong Lu

**Affiliations:** 1 School of Food and Pharmaceutical Engineering, Guizhou Institute of Technology, Guiyang 550025, China; 2 Center of Excellence in Fungal Research, Mae Fah Luang University, Chiang Rai 57100, Thailand; 3 School of Science, Mae Fah Luang University, Chiang Rai 57100, Thailand; 4 Engineering and Research Center for Southwest Biopharmaceutical Resource of National Education Ministry of China, Guizhou University, Guiyang, 550025, Guizhou Province, China; 5 Guizhou Industry Polytechnic College, Guiyang 550008, China; 6 Department of Botany and Microbiology, College of Science, King Saud University, P.O. Box 22452, Riyadh 11495, Saudi Arabia

**Keywords:** 3 new species, asexual morph, phylogeny, taxonomy

## Abstract

During our investigation of saprobic fungi in southwestern China, three micro-hyphomycetous fungi were isolated from dead wood in freshwater and terrestrial habitats in Guizhou Province. Phylogenetic analyses of ITS, LSU, and SSU sequences, performed using Maximum Likelihood and Bayesian Inference methods, confirmed that these isolates belong to *Kirschsteiniothelia*. Based on distinct morphological characteristics and molecular phylogenetic evidence, we describe three new species: *Kirschsteiniotheliaguizhouensis*, *K.weiningensis*, and *K.xishuiensis*. Furthermore, the effectiveness of three DNA markers for species-level identification within *Kirschsteiniothelia* was evaluated using Assemble Species by Automatic Partitioning (ASAP) analysis, which identified the ITS nucleotide sequences as the most reliable marker for species differentiation within the genus.

## ﻿Introduction

*Kirschsteiniothelia* was introduced by Hawksworth (1985) to accommodate the *Microtheliaincrustans*-group (Dothideales), with *K.aethiops* as the type species. Hawksworth (1985) and Barr (1987) placed the genus in Pleosporaceae. Subsequently, Barr (1993) suggested that this genus should be transferred to Pleomassariaceae based on its host, morphology, and asexual form. However, Schoch et al. (2006) recommended that this genus should have its own family, as the type species *K.aethiops* did not show close associations with Pleosporaceae based on their phylogenetic analyses. Boonmee et al. (2012) proposed a new family, Kirschsteiniotheliaceae to accommodate the taxa grouping with *K.aethiops*, and they transferred *K.elaterascus* to *Morosphaeria* (Morosphaeriaceae) and *K.maritima* to *Halokirschteiniothelia* (Mytilinidiaceae). *Kirschsteiniothelia* has been linked with the asexual genus *Dendryphiopsis*, as confirmed by several studies based on morphology and phylogeny (Hughes 1953; Schoch et al. 2009; Boonmee et al. 2012). Wijayawardene et al. (2014) proposed using *Kirschsteiniothelia* over *Dendryphiopsis* and designated *K.atra* as the type species. Subsequently, Hernandez-Restrepo et al. (2017) established a new order, Kirschsteiniotheliales, to accommodate this family where it is now included (Hyde et al. 2024).

The sexual morph of *Kirschsteiniothelia* is characterized by superficial, subglobose to globose, dark brown to black ascomata, with a peridium of *textura angularis*, cylindrical-clavate asci which are apically rounded with a small ocular chamber, and ellipsoidal, 1–2 septate, smooth-walled, olive brown to dark brown, dull green ascospores (Pem et al. 2024). The asexual morph has long, straight to slightly curved, septate, apically branched or unbranched, dark brown conidiophores, and broadly obovoid, fusiform or obclavate, reddish brown to dark brown or grayish brown, septate conidia (Hawksworth 1985; Boonmee et al. 2012; Mehrabi et al. 2017; Xu et al. 2023; Liu et al. 2024; Meng et al. 2024). Species of *Kirschsteiniothelia* are commonly found as saprobes from terrestrial and freshwater habitats in tropical or subtropical regions (Bao et al. 2018; Rodríguez-Andrade et al. 2019; Dong et al. 2020; Sun et al. 2021; Verma et al. 2021; Meng et al. 2024).

In this study, we collected four hyphomycetous samples of dead wood from freshwater and terrestrial habitats in Guizhou Province, China. Three new species, namely *Kirschsteiniotheliaguizhouensis*, *K.weiningensis* and *K.xishuiensis*, were identified based on morphological evidence and phylogenetic analyses of combined ITS, LSU, and SSU sequence data. This paper provides a polyphasic approach (molecular data, morphological characteristics and location information) for introducing the new species (Maharachchikumbura et al. 2021).

## ﻿Materials and methods

### ﻿Isolation and morphological observation

Decaying wood samples were randomly collected from Guizhou Xishui National Nature Reserve, Xishui County, within 28°9'17"–28°33'50"N, 105°55'50"–106°24'9"E; elevation 600–1,200 m, and Weining County, along the Wujiangyuan River (26°52'33"–26°57'50"N, 104°22'18"–104°25'43"E; elevation 2,000–2,100 m). The fresh specimens were stored in sterile, damp plastic containers at room temperature for approximately 15 to 20 days. The fresh samples were examined using stereo microscopes (SMZ 745 and SMZ 800N, Nikon, Tokyo, Japan), and their micro-morphological characteristics were observed using an ECLIPSE Ni compound microscope (Nikon, Tokyo, Japan).

The method for single spore isolation followed the procedure outlined by Senanayake et al. (2020). Purified cultures were maintained in an incubator at 28 °C under light conditions, and the morphological characteristics of the colonies were carefully observed and recorded. Dried specimens were deposited at the Herbarium of Kunming Institute of Botany, Chinese Academy of Sciences (Herb. HKAS), Kunming, China, as well as the Herbarium of the Guizhou Academy of Agriculture Sciences (GZAAS), Guiyang, China. The cultures were deposited in the Guizhou Culture Collection (GZCC), Guizhou, China. Index Fungorum (http://www.indexfungorum.org/names/names.asp) and Facesoffungi database (Jayasiri et al. 2015) numbers were obtained.

### ﻿DNA extraction, PCR amplification, and sequencing

Mycelia from 30-day-old cultures were scraped from PDA plates using sterile toothpicks and placed into 1.5 mL microcentrifuge tubes. DNA was extracted using the Ezup Column Fungi Genomic DNA Purification Kit, following the manufacturer’s instructions. PCR amplifications were performed for three loci: internal transcribed spacer (ITS), ribosomal large subunit rDNA (LSU), and ribosomal small subunit rDNA (SSU), using the primer pairs ITS5/ITS4 (White et al. 1990), LR0R/LR5 (Vilgalys and Hester 1990; Rehner and Samuels 1994) and NS1/NS4, respectively. The thermal cycling conditions for ITS, LSU, and SSU followed the procedures detailed by Ma et al. (2023). After PCR amplification, the products were analyzed using 1% agarose gel electrophoresis. Purification and sequencing of PCR products were carried out by Sangon Biotech (Shanghai) Co., Ltd. (Shanghai, China). The newly obtained sequences were deposited in the GenBank database (https://ncbi.nlm.nih.gov/WebSub/).

### ﻿Phylogenetic analyses

BioEdit version 7.0.5.3 (Hall 1999) was used to inspect the original sequences for quality, including checking base-calling errors, and potential contaminations or ambiguities in the nucleotide data. The SeqMan v. 7.0.0 (DNASTAR, Madison, WI, USA, Swindell and Plasterer 1997) was used to assemble forward and reverse sequences. The sequences used for phylogenetic analyses were obtained from GenBank (Table 1) and downloaded using the One-click Fungal Phylogenetic Tool (OFPT) (Zeng et al. 2023). For each locus, sequence alignments were performed using the online multiple alignment tool MAFFT version 7, and the resulting alignments were refined with the trimAl tool (Capella-Gutiérrez et al. 2009; Katoh and Standley 2013). The phylogenetic tree was constructed using the methods described by Su et al. (2022), which included Maximum Likelihood (ML) and Bayesian Inference (BI).

**Table 1.** The table below lists the taxa used in this study, with their respective GenBank accession numbers.

Taxon	Strain	GenBank Accessions		
		ITS	LSU	SSU
Kirschsteiniotheliaacutisporum*	MFLU 21-0127^T^	OP120780	ON980758	ON980754
*K.agumbensis*	NFCCI 5714^T^	PP029048	-	PP029049
K.aquatica*	MFLUCC 16-1685^T^	MH182587	MH182594	MH182618
K.arasbaranica*	IRAN 2509C	KX621986	KX621987	KX621988
K.arasbaranica*	IRAN 2508C^T^	KX621983	KX621984	KX621985
*K.atra*	DEN^T^	MG602687	-	-
*K.atra*	CBS 109.53	-	AY016361	AY016344
K.atra*	MFLUCC 16-1104	MH182583	MH182589	MH182615
K.atra*	S-783	MH182586	MH182595	MH182617
K.atra*	MFLUCC 15-0424	KU500571	KU500578	KU500585
K.atra*	GZCC 23-0731	PQ248940	PQ248936	PQ248932
K.bulbosapicalis*	GZCC 23-0732^T^	PQ248937	PQ248933	PQ248929
*K.cangshanensis*	MFLUCC 16-1350^T^	MH182584	MH182592	-
K.chiangmaiensis*	MFLU 23-0358^T^	OR575473	OR575474	OR575475
*K.crustaceum*	MFLU 21-0129^T^	MW851849	MW851854	
K.dendryphioides*	KUNCC 10431^T^	OP626354	PQ248935	PQ248931
*K.dendryphioides*	KUNCC 10499	PQ248938	-	-
K.dujuanhuensis*	KUNCC 22-12671^T^	OQ874971	OQ732682	OQ875039
K.dushanensis*	GZCC 19-0415^T^	OP377845	MW133830	MW134610
*K.ebriosa*	CBS H-23379^T^	-	LT985885	-
*K.ebriosa*	CBS 143842	-	LT985884	-
*K.emarceis*	MFLU 10-0037^T^	HQ441570	HQ441571	-
*K.esperanzae*	T.Raymundo 6581^T^	OQ877253	OQ880482	-
*K.extensum*	MFLU 21-0130^T^	MW851850	MW851855	-
*K.fluminicola*	MFLUCC 16-1263^T^	MH182582	MH182588	-
*K.guangdongensis*	ZHKUCC 22-0233^T^	OR164946	OR164974	-
K.guizhouensis*	**GZCC 24-0034^T^**	**PQ404852**	**PQ404856**	**PQ404859**
***K.guizhouensis***	**GZCC 24-0041**	**PQ404853**	-	**PQ404860**
K.inthanonensis*	MFLUCC 23-0277^T^	OR762773	OR762781	OR764784
*K.laojunensis*	KUN L 88727^T^	PP081651	PP081658	-
K.lignicola*	MFLUCC 10-0036^T^	HQ441567	HQ441568	HQ441569
K.longirostrata*	GZCC 23-0733^T^	PQ248939	PQ248934	PQ248930
K.longisporum*	UESTCC 24.0190^T^	PQ038266	PQ038273	PQ046108
K.nabanheensis*	HJAUP C2006	OQ023274	OQ023275	OQ023037
K.nabanheensis*	HJAUP C2004^T^	OQ023197	OQ023273	OQ023038
*K.phoenicis*	MFLU 18-0153	NR_158532	NG_064508	-
K.phoenicis*	MFLUCC 18-0216^T^	MG859978	MG860484	MG859979
K.pini*	UESTCC24.0131^T^	PP835321	PP835315	PP835318
K.puerensis*	ZHKUCC 22-0272	OP450978	OP451018	OP451021
K.puerensis*	ZHKUCC 22-0271^T^	OP450977	OP451017	OP451020
*K.ramus*	GZCC 23-0596^T^	NR_190260	NG_243331	-
K.rostrata*	MFLUCC 15-0619	KY697280	KY697276	KY697278
K.rostrata*	MFLU 15-1154^T^	NR_156318	NG_059790	NG_063633
*K.rostrata*	MFLUCC 16-1124	-	MH182590	-
*K.saprophytica*	MFLUCC 23-0275^T^	OR762774	OR762783	-
*K.saprophytica*	MFLUCC 23-0276	OR762775	OR762782	-
K.septemseptatum*	MFLU 21-0126^T^	OP120779	ON980757	ON980752
*K.sichuanensis*	UESTCC 24.0127^T^	PP785368	PP784322	-
*Kirschsteiniothelia* sp.*	KUNCC 23-13756	OR589303	OR600952	OR743201
*Kirschsteiniothelia* sp.	KUNCC 23-14559	OR589302	OR600951	-
*Kirschsteiniothelia* sp.*	KUNCC 23-13755	OR589301	OR600949	OR743199
*Kirschsteiniothelia* sp.	UTHSCSA DI22-44	ON191447	ON191450	-
*Kirschsteiniothelia* sp.	UTHSCSA DI22-45	ON191448	ON191449	-
*Kirschsteiniothelia* sp.	7020611638	MZ380314	MZ380317	-
*Kirschsteiniothelia* sp.	E38	MN912317	MN912273	-
*Kirschsteiniothelia* sp.	CSN604	MT813881	-	-
*Kirschsteiniothelia* sp.	CSN602	MT813880	-	-
*K.spatiosum*	MFLU 21-0128^T^	NR_187065	-	ON980753
*K.submersa*	S-481	-	MH182591	MH182616
*K.submersa*	S-601	MH182585	MH182593	-
K.submersa*	MFLUCC 15-0427^T^	KU500570	KU500577	KU500584
*K.tectonae*	MFLUCC 12-0050^T^	KU144916	KU764707	-
*K.tectonae*	MFLUCC 13-0470	KU144924	-	-
K.thailandica*	MFLUCC 20-0116^T^	NR_178154	NG_088170	NG_087878
K.thailandica*	MFLUCC 22-0020	ON878074	ON870387	ON870912
K.thailandica*	MFLU 20-0263	MT985633	MT984443	MT984280
K.thujina*	JF13210	KM982716	KM982718	KM982717
*K.vinigena*	CBS H-23378^T^	-	NG_075229	-
K.weiningensis*	**GZCC 24-0072^T^**	**PQ404851**	**PQ404855**	**PQ404858**
K.xishuangbannaensis*	ZHKUCC 22-0221	OP289563	OP303182	OP289565
K.xishuangbannaensis*	ZHKUCC 22-0220^T^	OP289566	OP303181	OP289564
K.xishuangbannaensis*	MFLUCC 23-0273	OR762770	OR762778	OR764781
K.xishuangbannaensis*	MFLUCC 23-0274	OR762769	OR762777	OR764780
K.xishuiensis*	**GZCC 24-0052^T^**	**PQ404850**	**PQ404854**	**PQ404857**
K.zizyphifolii*	MFLUCC 23-0270^T^	OR762768	OR762776	OR764779
*Pseudorobillardaeucalypti*	MFLUCC 12-0422	KF827451	KF827457	KF827463
*P.phragmitis*	CBS 398.61	MH858101	MH869670	EU754104

Note: “T” represents the ex-type strain. “-” indicates that no sequence data are available in GenBank. Newly generated sequences are represented in bold. “*” represents the analysis of ASAP strain.

The phylogenetic trees were edited using FigTree v1.4.0, and the final layout was completed using Adobe Photoshop 2018 and Adobe Illustrator 2021 (Adobe Systems, San Jose, CA, USA).

### ﻿Analysis of matrix partitions by Assemble Species by Automatic Partitioning (ASAP)

ASAP (Assemble Species by Automatic Partitioning) analysis was conducted using the ASAP online platform (https://bioinfo.mnhn.fr/abi/public/asap). The Kimura 2-Parameter model was selected to generate a list of partitions ranked by scores. To avoid unstable results due to gene deletion in some species, select “*” species for analysis.

## ﻿Results

### ﻿Phylogenetic analysis

**Figure 1.**
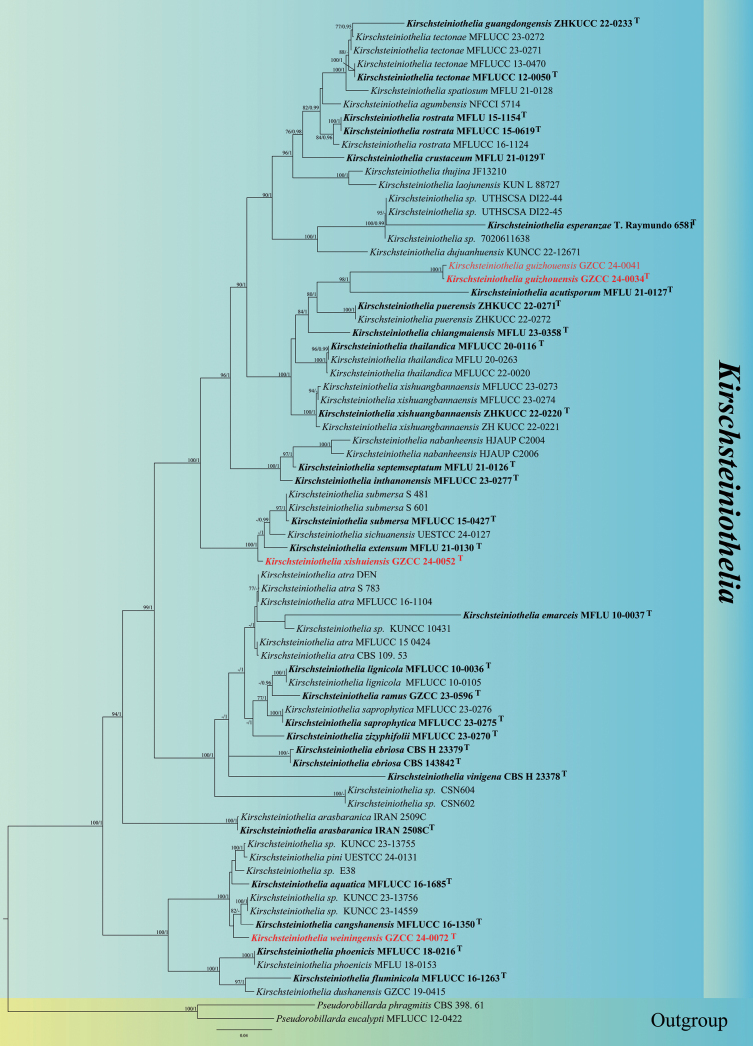
Maximum Likelihood (ML) majority rule consensus tree for the ITSLSU and SSU sequence data alignment of *Kirschsteiniothelia* and related taxa. *Pseudorobillardaeucalypti* (MFLUCC 12-0422) and *P.phragmitis* (CBS 398.61) are the outgroup taxa. ML bootstrap support values (MLB ≥ 75%) and Bayesian posterior probabilities (BYPP ≥ 0.95) are indicated below or above the nodes. Ex-type strains are in bold and marked with T, and the new species are in red.

The partial ITS, LSU and SSU nucleotide sequences were used to determine the phylogenetic positions of the new taxa, and the datasets consisted of 77 isolates representing 47 *Kirschsteiniothelia* species. *Pseudorobillardaeucalypti* (MFLUCC 12-0422) and *P.phragmitis* (CBS 398.61) were selected as the outgroup taxa. The concatenated sequence matrix includes ITS (1–506 bp), LSU (507–1,360 bp) and SSU (1,361–2,378 bp). The final ML optimization likelihood value of the best RAxML tree (Fig. 1) was -19027.817769, and the estimated base frequencies were as follows: A = 0.228043, C = 0.257324, G = 0.302209, T = 0.212423; substitution rates AC = 1.135651, AG = 2.447765, AT = 0.902710, CG = 1.148377, CT = 5.581689, GT = 1.000000. Gamma distribution shape parameter alpha is 0.279545. The concatenated ITS, LSU and SSU datasets were analyzed using ML and BI methods with similar tree topologies.

The resulting multi-gene phylogenetic tree confirmed that our newly obtained strains *Kirschsteiniotheliaguizhouensis* (GZCC 24-0034 and GZCC 24-0041), *K.xishuiensis* (GZCC 24-0052), and *K.weiningensis* (GZCC 24-0072) formed distinct clades within *Kirschsteiniothelia* (Fig. 1). Two isolates of *K.guizhouensis* (GZCC 24-0041 and GZCC 24-0034) clustered together and were sister to *K.acutisporum* (MFLU 21-0127) (100% MLB/1.00 BYPP). *Kirschsteiniotheliaxishuiensis* (GZCC 24-0052) formed a distinct clade basal to *K.submersa* (S-481, S-601, and MFLUCC 15-0427), *K.sichuanensis* (UESTCC 24.0127) and *K.extensum* (MFLU 21-0130) (100% MLB/1.00 BYPP). While *K.weiningensis* (GZCC 24-0072) grouped with *K.cangshanensis* (MFLUCC 16-1350) and *Kirschsteiniothelia* sp. (KUNCC 23-13756 and KUNCC 23-14559), but in a distinct lineage (96% MLB/0.98 BYPP).

### ﻿Assemble species by Automatic Partitioning (ASAP) results

Three single-locus (ITS, LSU, SSU) datasets, comprising 41 strains were used for analysis. The ASAP analysis of ITS region identified 30 distinct groups within *Kirschsteiniothelia*. Similarly, the LSU region was classified into 27 groups, while the SSU region was divided into 22 groups.

In the ASAP analysis, *Kirschsteiniothelialongisporum* (UESTCC 24.0190), *K.pini* (UESTCC 24-0131), *Kirschsteiniothelia* sp. (KUNCC 23-13756 and KUNCC 23-13755), and *K.weiningensis* (GZCC 24-0072) grouped together based on the SSU dataset. However, they were divided into five distinct groups in the ITS dataset and three groups in the LSU dataset. *Kirschsteiniotheliathailandica* (MFLUCC 20-0116, MFLUCC 22-0020, and MFLU 20-0263) and *K.xishuangbannaensis* (ZHKUCC 22-0221, ZHKUCC 22-0220, MFLUCC 23-0273, and MFLUCC 23-0274) were treated as a single group based on the SSU dataset. However, in the ITS, LSU, and combined datasets, they were divided into two distinct groups. *Kirschsteiniotheliaxishuiensis* (GZCC 24-0052) and *K.submersa* (MFLUCC 15-0427) grouped together in the SSU dataset but were identified as separate species in the ITS and LSU datasets. *Kirschsteiniotheliaguizhouensis* (GZCC 24-0034) consistently appeared as a single group across all single-marker analyses.

**Figure 2.**
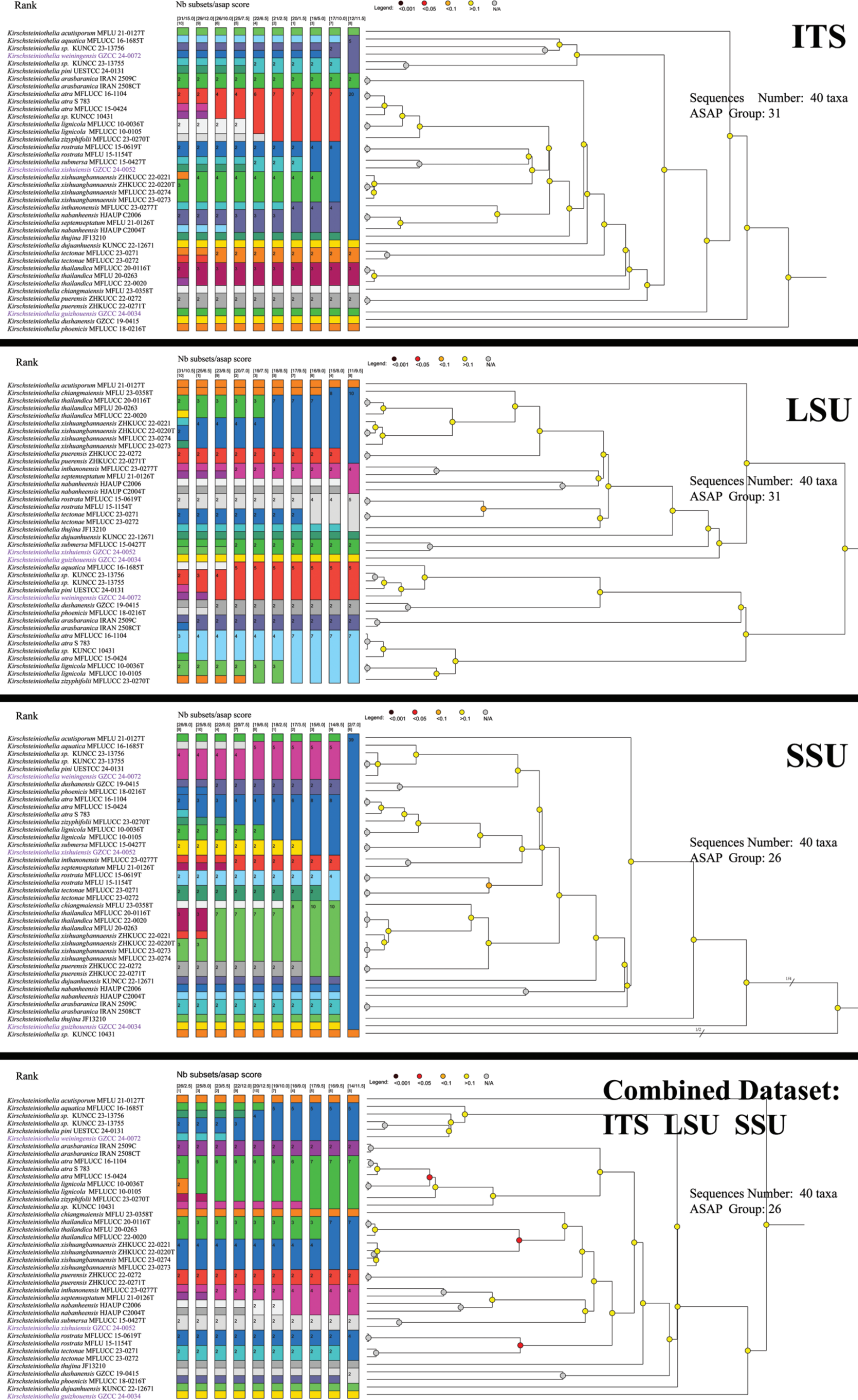
Dendrogram from ASAP analysis based on three datasets including ITS, LSU and SSU markers. The results of species delimitation are indicated by different color bars. The new species introduced in this study are purple.

Therefore, the ITS is currently considered the most reliable marker for identifying *Kirschsteiniothelia* taxa at the species level, following the principle that “the smaller the ASAP score, the better” (Puillandre et al. 2021).

### ﻿Taxonomy

#### ﻿ Kirschsteiniothelia guizhouensis

Taxon classificationFungiKirschsteiniothelialesKirschsteiniotheliaceae

X.J. Xiao, Y.Z. Lu & K.D. Hyde sp. nov.

FF3ABDC9-3EDA-5C50-8A14-659E4D7344DF

Index Fungorum: IF903149

Facesoffungi Number: FoF16776

Fig. 3

##### Etymology.

Referring to the collecting location at Guizhou Province in China.

##### Holotype.

HKAS 139503.

##### Description.

***Saprobic*** on submerged decaying wood in a freshwater habitat. ***Sexual morph***: Undetermined. ***Asexual morph*: *Colonies*** on natural substrate effuse, dark brown to black with white tip, hairy. ***Mycelium*** immersed, composed of brown to dark brown, branched, septate, smooth hyphae. ***Conidiophores*** 195–477 × 11–16 μm (x̄ = 307 × 13.5 µm, n = 20), macronematous, mononematous, erect, straight to slightly curved, apically branched, cylindrical, tapering towards the apex, dark brown, multi-septate, thick-walled. ***Conidiogenous cells*** 5.5–16 × 4.5–8 μm (x̄ = 11 × 6 µm, n = 30), monoblastic, integrated and discrete, terminal at the apex of the stem and branches, subcylindrical, light to dark brown. ***Conidia*** 36.5–65 × 8–16.5 μm (x̄ = 50 × 12.5 µm, n = 20), acrogenous, solitary, dry, olivaceous brown to brown, pale brown to hyaline at the apex, obclavate, sometimes apical cell swollen to subglobose, rostrate, straight or curved, truncate at base, septate, slightly constricted at septa, with an apical, hyaline, mucilaginous sheath, 14–39 × 14–36 μm (x̄ = 23 × 23 µm, n = 20).

##### Cultural characteristics.

Conidia germinating on PDA medium within 24 h and germ tube produced from apex. *Colonies* on PDA medium reaching to 19.5 mm diam in 13 days at 28 °C in natural light, circular, dense, mycelium slightly aerial, with entire edge, dark green from above and below.

**Figure 3.**
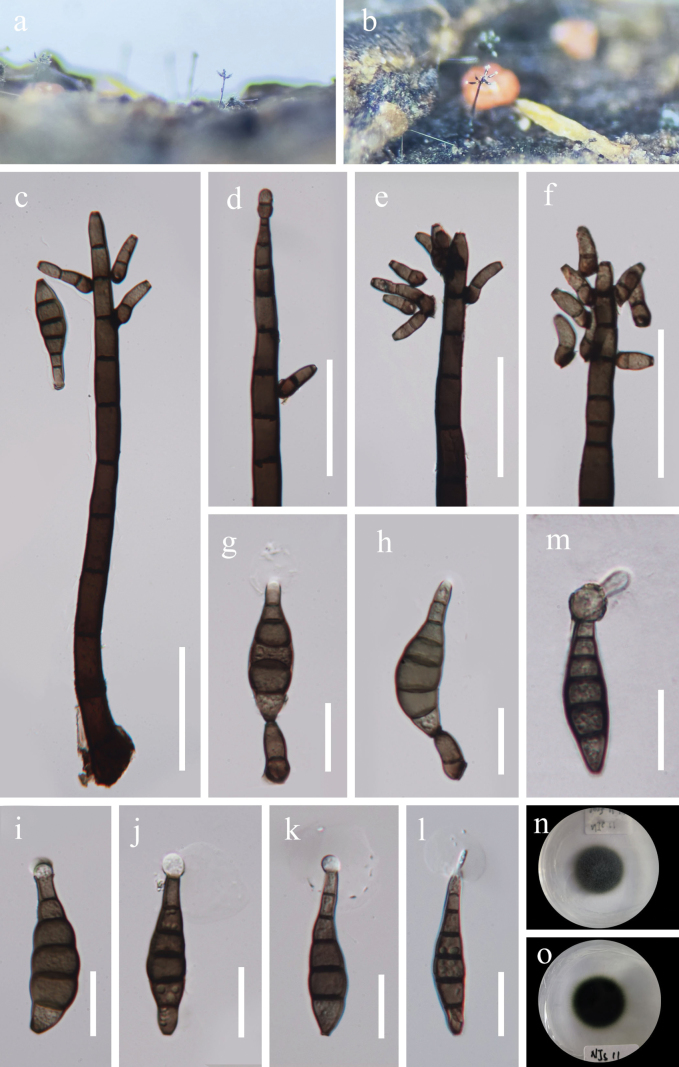
*Kirschsteiniotheliaguizhouensis* (HKAS 132503, holotype) **a, b** colonies on dead wood **c** conidiophore **d–f** conidiogenous cells **g, h** conidiogenous cells and conidia **i–l** conidia (**g, j–l** sheath is marked with a yellow arrow) **m** a germinated conidium **n, o** colonies on PDA (**n** upper view **o** lower view). Scale bars: 50 μm (**c–f**); 20 μm (**g–m**).

##### Material examined.

China • Guizhou Province, Xishui County, Guizhou Xishui National Nature Reserve, 28°9'17"N, 105°55'50"E, on decaying wood in freshwater, 2 October 2023, Xingjuan Xiao, NJS11 (HKAS 139503, holotype), ex-type living strain GZCC 24-0034; • Ibid., NJS36.1 (HKAS 139504, paratype), living strain GZCC 24-0041.

##### Notes.

In the phylogenetic tree, our strains (GZCC 24-0034 and GZCC 24-0041) cluster together, forming a sister clade to *Kirschsteiniotheliaacutisporum* (MFLU 21-0127) (Fig. 1). A comparison of nucleotides between *K.guizhouensis* (GZCC 24-0034) and *K.acutisporum* showed similarity rates in the ITS 77% (407/528 bp, 36 gaps), LSU 93% (785/847 bp, 13 gaps), and SSU 96% (818/849 bp, 2 gaps), indicating significant differences between the two species. Morphologically, our species exhibits distinct characteristics compared to *K.acutisporum*, including smaller conidia with a mucilaginous sheath (36.5–65 × 8–16.5 µm vs. 75–120 × 10.5–19.5 μm), and longer conidiophores which are branched at the apex (195–477 × 11–16 μm vs. 180–260 × 7–12.5 μm) (Jayawardena et al. 2022). Therefore, we propose *K.guizhouensis* as a new species.

#### ﻿ Kirschsteiniothelia xishuiensis

Taxon classificationFungiKirschsteiniothelialesKirschsteiniotheliaceae

X.J. Xiao, Y.Z. Lu & K.D. Hyde sp. nov.

8994F268-0382-5C72-9B79-3DF8AECE9C3C

Index Fungorum: IF903150

Facesoffungi Number: FoF16777

Fig. 4

##### Etymology.

Referring to the collecting location at Xishui District in China.

##### Holotype.

HKAS 132145.

##### Description.

***Saprobic*** on decaying wood in a terrestrial habitat. ***Sexual morph***: Undetermined. ***Asexual morph*: *Colonies*** on natural substrate effuse, dark brown to black, hairy. ***Mycelium*** immersed, composed of brown to dark brown, branched, septate, smooth hyphae. ***Conidiophores*** 170–280 × 8–13 µm (x̄ = 207 × 10.5 µm, n = 20), macronematous, mononematous, erect, straight to slightly curved, unbranched, cylindrical, dark brown, multi-septate, thick-walled. ***Conidiogenous cells*** 13–17.5 × 5.5–9 µm (x̄= 15.5 × 7 μm, n = 20), monoblastic, integrated, terminal, cylindrical, mid to dark brown. ***Conidia*** 35.5–67.5 × 11–20 µm (x̄= 48.5 × 15.5 μm, n = 20), acrogenous, solitary, dry, olivaceous brown to soot brown, paler at apex, obclavate, rostrate, straight or curved, truncate at base, septate, constricted at septa, with an apical, hyaline, mucilaginous sheath, 13–39 × 14–35 µm (x̄= 24 × 23 μm, n = 20).

**Figure 4.**
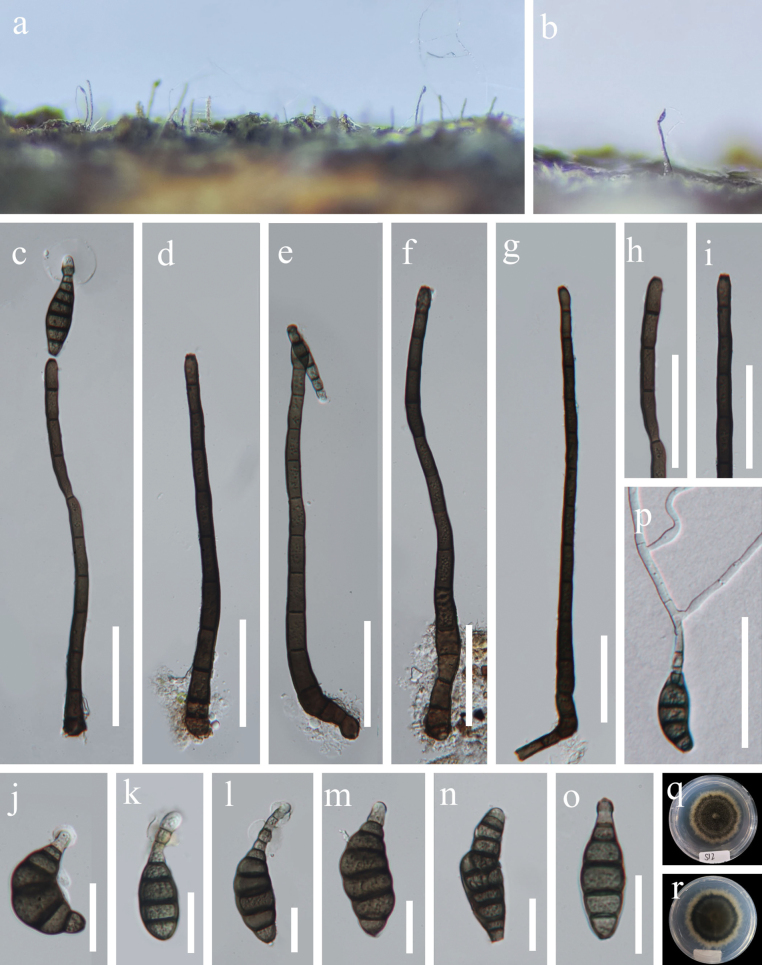
*Kirschsteiniotheliaxishuiensis* (HKAS 132145, holotype) **a, b** colonies on dead wood **c–g** conidiophores, conidiogenous cells and conidia **h, i** conidiogenous cells **j–o** conidia (**c, k, l** sheath is marked with a yellow arrow) **p** a germinated conidium **q, r** colonies on PDA (**q** upper view **r** lower view). Scale bars: 50 μm (**c–i, p**); 20 μm (**j–o**).

##### Cultural characteristics.

Conidia germinating on PDA medium within 12 h and germ tube produced from truncate end. *Colonies* on PDA medium reaching to 35 mm diam in 35 days at 28 °C in natural light, circular, dense, mycelium slightly aerial, with irregular margin, grayish brown at center, yellowish brown at outer ring from above and below.

##### Material examined.

China • Guizhou Province, Xishui County, Guizhou Xishui National Nature Reserve, 28°33'50"N, 106°24'9"E, on decaying wood in a forest, 3 October 2023, Xingjuan Xiao, S12 (HKAS 132145, holotype), ex-type living strain GZCC 24-0052.

##### Notes.

Phylogenetically, *Kirschsteiniotheliaxishuiensis* (GZCC 24-0052) formed a distinct clade basal to *K.submersa* (S-481, S-601, and MFLUCC 15-0427), *K.sichuanensis* (UESTCC 24.0127) and *K.extensum* (MFLU 21-0130) (Fig. 1). BLASTN analysis of *K.xishuiensis* (GZCC 24-0052) reveals 93% identity (452/483, 7 gaps) to *K.submersa* (S-601) in the ITS gene region. Similarly, *K.xishuiensis* shows 93% identity (410/443, 4 gaps) to *K.sichuanensis* (UESTCC 24.0127) and 93% identity (463/497, 5 gaps) to *K.extensum* (MFLU 21-0130) when analyzed using ITS. Morphologically, *K.xishuiensis* is similar to *K.extensum* in having straight to slightly curved, unbranched, cylindrical, dark brown, multi-septate conidiophores, but differs from *K.extensum* in having shorter and wider conidia with a gelatinous rounded sheath at the apex (35.5–67.5 × 11–20 µm vs. 45–120 × 5–12 μm), and larger conidiophores (170–280 × 8–13 μm vs. 80–230 × 6.5–9.5 μm) (Jayawardena et al. 2022). Therefore, we propose *K.guizhouensis* as a new species based on both morphology and molecular data.

#### ﻿ Kirschsteiniothelia weiningensis

Taxon classificationFungiKirschsteiniothelialesKirschsteiniotheliaceae

X.J. Xiao, Y.Z. Lu & K.D. Hyde sp. nov.

134F1671-EEB3-5FAD-AB63-36099DCA342C

Index Fungorum: IF903151

Facesoffungi Number: FoF16778

Fig. 5

##### Etymology.

Referring to the collecting location at Weining District in China.

##### Holotype.

HKAS 132143.

##### Description.

***Saprobic*** on decaying wood in a freshwater habitat. ***Sexual morph***: Undetermined. ***Asexual morph*: *Colonies*** on natural substrate effuse, dark brown to black, hairy. ***Mycelium*** immersed, composed of brown to dark brown, branched, septate, smooth hyphae. ***Conidiophores*** 75–125 × 5–10 μm (x̄ = 97 × 7 µm, n = 20), macronematous, mononematous, erect, straight to slightly curved, unbranched, cylindrical, brown to dark brown, multi-septate, thick-walled. ***Conidiogenous cells*** 10–25 × 5–8 µm (x̄ = 15 × 6 μm, n = 20), holoblastic, monoblastic, integrated, terminal, cylindrical, mid to dark brown, percurrently proliferating. ***Conidia*** 20–45 × 6–10 µm (x̄ = 37 × 8 μm, n = 20), acrogenous, solitary, dry, pale brown to brown, obclavate, rostrate, straight or slightly curved, truncate at base, septate, slightly constricted at the septa, with a gelatinous sheath at apex.

**Figure 5.**
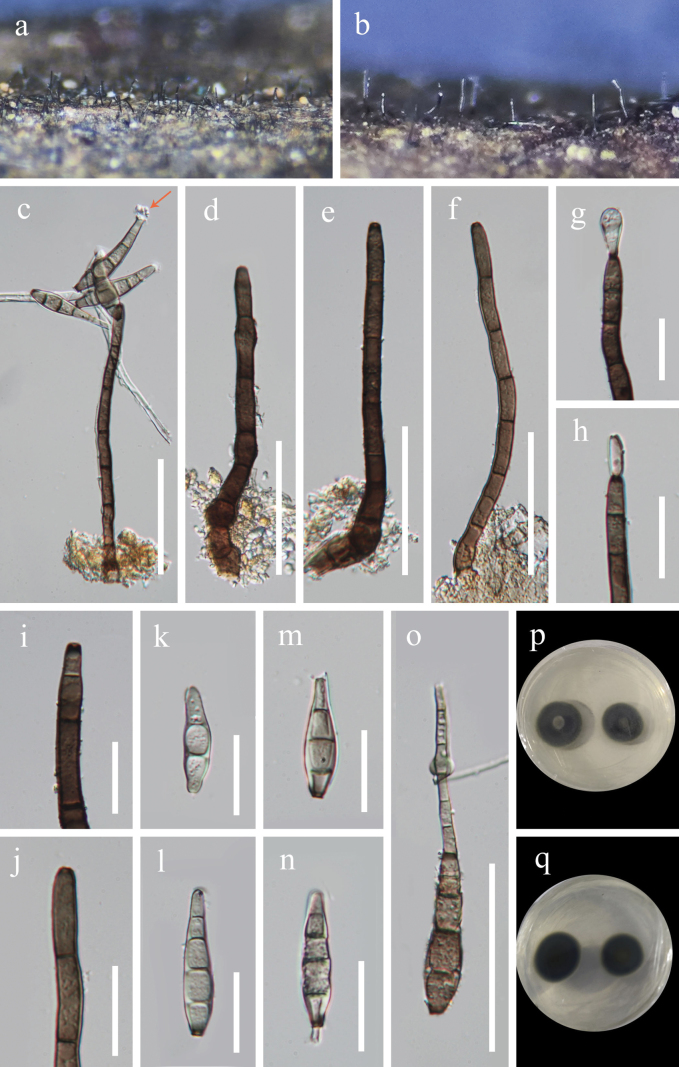
*Kirschsteiniotheliaweiningensis* (HKAS 132143, holotype) **a, b** colonies on dead wood **c–f** conidiophores, conidiogenous cells and conidia **g–j** conidiogenous cells and young conidia **k–o** conidia (**c, m** sheath is marked with a yellow arrow) **p, q** colonies on PDA (**p** upper view **q** lower view). Scale bars: 50 μm (**c–f, o**); 20 μm (**g–n**).

##### Cultural characteristics.

Conidia germinating on PDA medium within 24 h and germ tube produced from the truncate base. *Colonies* on PDA medium reaching to 21 mm diam in 24 days at 28 °C in natural light, circular, dense, mycelium slightly aerial, with raised center and rounded edge, grayish green to dark green from above and below.

##### Material examined.

China • Guizhou Province, Weining County, Wujiangyuan river, 26°52'33"N, 104°22'18"E, on decaying wood in a freshwater habitat, 2 August 2023, Xingjuan Xiao, WJY23 (HKAS 132143, holotype), ex-type living strain GZCC 24-0072.

##### Notes.

Phylogenetically, *Kirschsteiniotheliaweiningensis* (GZCC 24-0072) grouped with *K.cangshanensis* (MFLUCC 16-1350) and *Kirschsteiniothelia* sp. (KUNCC 23-13756 and KUNCC 23-14559), but in a distinct lineage (Fig. 1). *Kirschsteiniotheliaweiningensis* and *K.cangshanensis* are both sporidesmium-like taxa and share highly similar characteristics, which render their differentiation based solely on morphology a challenge. This is a common occurrence among many sporidesmium-like taxa (Su et al. 2016). Nevertheless, ITS comparison reveals that *K.weiningensis* (GZCC 24-0072) exhibits 92% identity (684/740,14 gaps) and 93% identity (474/502, 2 gaps) to *K.cangshanensis* (MFLUCC 16-1350) and *Kirschsteiniothelia* sp. (KUNCC 23-13756), respectively. Therefore, we propose *K.weiningensis* as a new species.

## ﻿Discussion

According to Index Fungorum (accessed on 15 December 2024, https://www.indexfungorum.org/Names/Names.asp), 55 species are currently listed under *Kirschsteiniothelia* and thus it is a speciose genus. Among these, *K.elaterascus* and *K.maritima* have been reclassified (Boonmee et al. 2012), while *K.incrustans* and *K.aethiops* are considered synonyms of *K.atra* (Wijayawardene et al. 2014). Additionally, recently introduced species, such as *K.agumbensis*, *K.arbuscula*, *K.binsarensis*, *K.biseptata*, *K.fasciculari*, *K.goaensis*, and *K.longisporum* have not been released in the Index Fungorum database (Jin et al. 2024; Sruthi et al. 2024). Thus, *Kirschsteiniothelia* currently comprises 58 species, of which only 38 have available sequence data. This confirms the assumption of Bhunjun et al. (2022) that speciose genera are likely to contain further new taxa.

Species of this genus have been reported in diverse regions, including Africa, Canada, China, India, Iran, Japan, Mexico, Spain, Switzerland, Thailand and the United States (Zhang and Fournier 2015; Li et al. 2016; Mehrabi et al. 2017; Bao et al. 2018; Rodríguez-Andrade et al. 2019; Sun et al. 2021; Raymundo et al. 2023; Xu et al. 2023; Louangphan et al. 2024; Meng et al. 2024; Sruthi et al. 2024; Tian et al. 2024). In China, 21 *Kirschsteiniothelia* species have been reported with most species being its asexual morphs (three sexual species and 18 asexual species), with nine species in Yunnan Province, three species in Sichuan Province, four species in Hainan Province, two species in Taiwan Province, and one species each in Guizhou Province and Guangdong Province. The generic type, *Kirschsteiniotheliaatra* has been discovered in Guizhou and Yunnan Provinces (Chen et al. 2006; Su et al. 2016; Bao et al. 2018; Hyde et al. 2023; Liu et al. 2023; Senanayake et al. 2023; Yang et al. 2023; Zhang et al. 2023; Jin et al. 2024; Meng et al. 2024; Tang et al. 2024; Tian et al. 2024). Information on the morphological characteristics of the asexual species reported in China is shown in Table 2.

**Table 2.** Synopsis of the morphological characteristics of asexual taxa of *Kirschsteiniothelia* species reported from China.

No.	Species	Distribution	Habitat	Host	Conidiophores	Conidiogenous cells	Conidia	References
1	*Kirschsteiniotheliaaquatica*	Yunnan Province	Freshwater	Dead wood	Unbranched, cylindrical, dark brown, 114–151 × 7–8 μm.	Monoblastic, cylindrical, dark brown.	Obclavate, smooth, septate, dark brown, 35–46 × 7.5–8.5 μm.	Bao et al. (2018)
2	*K.atra*	Yunnan Province, Guizhou Province	Freshwater, terrestrial	Dead wood, *Edgeworthiachrysantha*	Branched, cylindrical, 5–10-septate, dark brown, 245–355 × 8–10 μm.	Doliiform or lageniform, pale brown or subhyaline.	Cylindrical, smooth, 3–4 septate, brown, 54–63 × 14–18 μm.	Su et al. (2016), Tang et al. (2024)
3	*K.bulbosapicalis*	Hainan Province	Terrestrial	Dead wood	Unbranched, smooth, brown to dark brown, 58–128 × 7.5–12.5 μm.	Cylindrical, brown to dark brown, 6–17 × 7–10.5 μm.	Cylindrical, ovoid to obclavate, rostrate, smooth, septate, olivaceous to reddish-brown to dark brown, with sheath, 118–236.5 × 15–27 μm.	Tang et al. (2024)
4	*K.cangshanensis*	Yunnan Province	Freshwater	Dead wood	Unbranched, cylindrical, pale brown, 105.5–135.5 × 6–8 μm.	Monoblastic, cylindrical, pale brown.	Obclavate, septate, pale brown to brown, with sheath, 33–43 × 7.5–8.5 μm.	Bao et al. (2018)
5	*K.dendryphioides*	Yunnan Province	Freshwater	Dead wood	Branched, smooth, brown to dark brown, 179–467 × 4.5–8 μm.	Cylindrical, doliiform, pale brown to brown, 9–19 × 4–8 μm.	Cylindrical, oblong and occasionally clavate, smooth, guttulate, 2–4 septate, brown, 30–55 × 9–13.5 μm.	Tang et al. (2024)
6	*K.dushanensis*	Guizhou Province	Freshwater	Dead wood	Unbranched, cylindrical, verrucose, septate, dark brown, 160–307 × 6.5–13 μm.	Monoblastic, cylindrical or doliform, brown, 9–26 × 3–7 μm.	Rostrate, smooth, 5–11 septate, olivaceous brown to soot brown, with sheath, 62–81 × 12.5–18 μm.	Yang et al. (2023)
7	*K.fluminicola*	Yunnan Province	Freshwater	Dead wood	Unbranched, cylindrical, smooth, dark brown to black, 209–286 × 7–9 μm.	Monoblastic, cylindrical, dark brown.	Solitary to short-catenate, obclavate, rostrate, multi-septate, subhyaline to dark brown, guttulate, 47.5–86.5 × 8–10 μm.	Bao et al. (2018)
8	*K.guangdongensis*	Guangdong Province	Terrestrial	Submerged wood	Unbranched, cylindrical, septate, dark brown, 250–350 × 10–18 μm.	Monoblastic, cylindrical to ampulliform, dark brown, 15–18 × 9–12 μm.	Elongated, flask-shaped, smooth, 13 septate, blackish brown to black, with sheath, 290-300 um long, 42-50 um wide at base, 20-22 um wide at apex.	Senanayake et al. (2023)
9	*K.guizhouensis*	Guizhou Province	Freshwater	Dead wood	Branched, cylindrical, multi-septate, thick-walled, dark brown, 195–477 × 11–16 μm.	Monoblastic, branches, subcylindrical, light to dark brown, 5.5–16 × 4.5–8 μm.	Obclavate, rostrate, septate, olivaceous brown to brown, with sheath, 36.5–65 × 8–16.5 μm.	This study
10	*K.longirostrata*	Hainan Province	Terrestrial	Dead wood	Unbranched, smooth, brown to dark brown, 80–252 × 4.5–9.5 μm.	Cylindrical, pale brown to brown, 6.5–16 × 5–9 μm.	Cylindrical, obpyriform to obclavate, rostrate, smooth, septate, pale brown to brown, with sheath, 36.5–109 × 8–16 μm.	Tang et al. (2024)
11	*K.longisporum*	Sichuan Province	Terrestrial	*Pinustaeda*	Branched, solitary or fasciculate, erect, cylindrical, septate, verruculose, dark brown to black, 115–285 × 6.5–14 μm.	Cylindrical, verruculose, dark brown.	Cylindrical, obclavate, elongated, thick-walled, 3–15 septate, verruculose, brown, 35–130 × 8.5–15 µm.	Tian et al. (2024)
12	*K.nabanheensis*	yunnan Province	Terrestrial	Dead wood	Irregular or subscorpioid branched, cylindrical, smooth, septate, black, brown to brown, 320–588 × 8–12 μm.	Monotretic, cylindrical or doliiform, brown to dark brown, 20–24 × 4–6 μm.	Obclavate or fusiform, 3–7 septate, smooth, dark brown to brown, 32–112 × 8–12 μm.	Liu et al. (2023)
13	*pini*	Sichuan Province	Terrestrial	*Pinus* sp.	Unbranched, cylindrical, 3–8 septate, smooth, brown or dark brown, 69–124 × 3.5–7 µm.	Monoblastic, cylindrical, pale brown, 15–21 × 3–5 µm.	Obclavate, 3–6 septate, brown, 22–45 × 5–10 µm.	Jin et al. (2024)
14	*K.puerensis*	Yunnan Province	Terrestrial	*Coffea* sp.	Unbranched, solitary or caespitose, cylindrical, smooth, 7–15 septate, dark brown, 100–250 × 5–12 μm.	Dark brown to black, smooth, 15–25 × 5–10 μm.	Obclavate, 5–12 septate, pale brown to brown, a hyaline sheath (some two globose sheaths), 60–140 × 5–20 μm.	Hyde et al. (2023)
15	*K.ramus*	Hainan Province	Freshwater	Dead wood	Simple or mostly apically branched, cylindrical, septate, brown, 102–248 × 5–11 μm.	Monotretic, branches, pale brown to brown, 18–27 × 6.5–9 μm.	Cylindrical, 2–3 septate, verruculose, brown, 42–56 × 15–22 μm.	Zhang et al. (2023)
16	*K.rostrata*	Yunnan Province	Freshwater	Dead wood	Unbranched, smooth, septate, brown to dark brown, 90–120 × 7.5–8.5 μm.	Monoblastic, cylindrical or lageniform, smooth, mid to dark brown.	Obclavate, rostrate, smooth, 6–17 septate, olivaceous brown to brown, 77.5–108.5 × 17.5–20.5 µm.	Bao et al. (2018)
17	*sichuanensis*	Sichuan Province	Terrestrial	Dead wood	Unbranched, cylindrical, 4–8 septate, smooth, brown or dark brown, 82–194 × 5–10 μm.	Monoblastic, cylindrical, pale brown, 10–22 × 6–9 μm.	Obclavate, 2–7 septate, smooth, brown, 34–54 × 8–14 μm.	Jin et al. (2024)
18	*K.submersa*	Yunnan Province	Freshwater	Dead wood	Unbranched, cylindrical, smooth, multi-septate, blackish to brown, 220–280 × 6–7 μm.	Monoblastic, cylindrical, pale brown.	Obclavate, 4–6 septate, smooth, brown to pale brown, 37.5–51.5 × 8.5–9.5 μm.	Su et al. (2016)
19	*K.weiningensis*	Guizhou Province	Freshwater	Dead wood	Unbranched, cylindrical, multi-septate, brown to dark brown, 74–122 × 5–9 μm.	Monoblastic, cylindrical, mid to dark brown, 9–23 × 4–8 μm.	Obclavate, rostrate, septate, pale brown to brown, 20–45 × 6–10 μm wide.	This study
20	*K.xishuangbannaensis*	Yunnan Province	Terrestrial	*Heveabrasiliensis*	Septate, brown to dark brown, 35–150 × 5–15 μm.	Cylindrical or lageniform, smooth, brown to dark brown, 10–50 × 5–10 μm.	Obclavate, rostrate, some have guttulate, 3–8 septate, yellow-brown to brown, with sheaths, 30–150 × 5–20 μm.	Xu et al. (2023)
21	*K.xishuiensis*	Guizhou Province	Terrestrial	Dead wood	Unbranched, cylindrical, multi-septate, dark brown, 170–280 × 8–13 μm.	Monoblastic, cylindrical, mid to dark brown, 13–17.5 × 5.5–9 μm.	Obclavate, rostrate, septate, olivaceous brown to soot brown, with sheath, 35.5–67.5 × 11–20 μm.	This study

In this study, the ASAP tool (Puillandre et al. 2021) was used to determine the most informative locus for species delimitation within *Kirschsteiniothelia*. The ITS gene regions provided the most reliable species-level identification, followed by LSU and SSU (Fig. 2). The ITS dataset exhibited similarities with the LSU dataset group, and the ITS had a lower ASAP score. The differences in ITS among species in this genus are more pronounced. These results suggest that ITS is the most suitable for species identification in this genus.

In recent research, Nishi et al. (2018) reported the first human case of *Kirschsteiniothelia* infection, which occurred in a patient with pre-existing non-infectious bursitis of the ankle. Due to the limited number of studies on mycoses, public awareness of fungal infections remains low (Denning 2016; Bongomin et al. 2017; Pegorie et al. 2017; Boniche et al. 2020). Therefore, future research should prioritize the study of fungal diseases. Additionally, attention to safety and prevention of fungal infections is crucial, especially for fungal taxonomists.

## Supplementary Material

XML Treatment for Kirschsteiniothelia guizhouensis

XML Treatment for Kirschsteiniothelia xishuiensis

XML Treatment for Kirschsteiniothelia weiningensis

## ﻿Additional information

### Author ORCIDs

Xing-Juan Xiao https://orcid.org/0009-0003-8769-4534

Ning-Guo Liu https://orcid.org/0000-0002-9169-2350

Jian Ma https://orcid.org/0009-0008-1291-640X

Li-Juan Zhang https://orcid.org/0000-0002-3234-6757

Fatimah Al-Otibi https://orcid.org/0000-0003-3629-5755

Kevin D. Hyde https://orcid.org/0000-0002-2191-0762

Yong-Zhong Lu https://orcid.org/0000-0002-1033-5782

### Data availability

All of the data that support the findings of this study are available in the main text.
